# Forms of Violent Behavior in Patients with Mental Disorders at the University Psychiatric Clinic Ljubljana from 2018 to 2021 Using the OAS/BVC Scale Assessment—A Descriptive Study

**DOI:** 10.2478/sjph-2026-0021

**Published:** 2026-09-01

**Authors:** Rok Hatze, Maja Šikić Pogačar, Jožica Peterka Novak, Branko Bregar

**Affiliations:** University Psychiatric Clinic Ljubljana, Chengdujska 45, 1260 Ljubljana, Slovenia; Faculty of Medicine, University of Maribor, Taborska ulica 8, 2000 Maribor, Slovenia; University Psychiatric Clinic Ljubljana, Chengdujska 45, 1260 Ljubljana, Slovenia, retired; National Institute of Public Health, Trubarjeva cesta 2, 1000 Ljubljana, Slovenia; Angela Boškin Faculty of Health Care, Spodnji Plavž 3, 4270 Jesenice, Slovenia

**Keywords:** Aggression, Psychiatric hospitals, Inpatients, Mental disorders, Overt Aggression Scale (OAS), Brøset Violence, Checklist (BVC), Risk assessment, agresija, psihiatrične bolnišnice, hospitalizirani bolniki, duševne motnje, lestvica očitne agresije (angl. Overt Aggression Scale), kontrolni seznam nasilja Brøset (angl. Brøset Violence Checklist), ocena tveganja

## Abstract

**Introduction:**

The high incidence of violence directed at healthcare workers is a major public health concern. The purpose of the study was to investigate the incidence of various forms of aggression at the University Psychiatric Clinic Ljubljana from 2018 to 2021, stratified by perpetrator gender, time of occurrence, and ward type.

**Methods:**

Data were collected using completed standardised forms (the OAS and BVC scales) during 2018–2021. A total of 4,392 episodes of various forms of violent behaviour were detected. Descriptive and bivariate statistics were performed using IBM SPSS. The level of statistical significance was set at p < 0.05.

**Results:**

The total aggression score was significantly higher in men (t = 8.471, p < 0.001). Wards differed in the forms of aggressive behaviour (p < 0.05). The total aggression score (AS score) was significantly higher during the evening shift than during the morning shift (p < 0.001) or the night shift (p = 0.037). A positive correlation was found between OAS and BVC scores (r = 0.672, p < 0.001).

**Conclusions:**

Our findings are consistent with previous international research, confirming that violence remains a common challenge in psychiatric inpatient settings. The positive association between BVC and OAS scores supports the complementary use of both instruments for violence risk assessment and incident evaluation. In line with previous research, the results support recommendations that violence prevention should extend beyond assessment tools and staff training to include a culture of safety and organisational commitment to preventing and managing aggression.

## Izvleček

**Uvod:**

Visoka pojavnost nasilja nad zdravstvenimi delavci predstavlja pomemben javnozdravstveni problem. Namen raziskave je bil preučiti pojavnost različnih oblik agresivnega vedenja na Univerzitetni psihiatrični kliniki Ljubljana v obdobju 2018–2021 glede na spol povzročitelja, čas dogodka in vrsto oddelka.

**Metode:**

Podatki so bili zbrani s pomočjo izpolnjenih standardiziranih obrazcev (lestvici OAS in BVC) v obdobju 2018–2021. Skupno je bilo zaznanih 4.392 epizod različnih oblik nasilnega vedenja. Analiza podatkov je bila izvedena z uporabo opisne in bivariatne statistike v programu IBM SPSS. Meja statistične značilnosti je bila določena pri p < 0,05.

**Rezultati:**

Skupni rezultat agresivnosti je bil statistično značilno višji pri moških (t = 8,471; p < 0,001). Med oddelki so bile ugotovljene statistično značilne razlike glede oblik agresivnega vedenja (p < 0,05). Skupni rezultat agresivnosti (AS score) je bil statistično značilno višji v popoldanski izmeni v primerjavi z dopoldansko (p < 0,001) in nočno izmeno (p = 0,037). Med rezultatoma lestvic OAS in BVC je bila ugotovljena pozitivna povezanost (r = 0,672; p < 0,001).

**Zaključki:**

Naše ugotovitve so skladne s predhodnimi mednarodnimi raziskavami, ki potrjujejo, da nasilje ostaja pogost izziv v psihiatrični bolnišnični obravnavi. Pozitivna povezanost med rezultatoma lestvic BVC in OAS podpira njuno komplementarno uporabo pri ocenjevanju tveganja za nasilje in vrednotenju agresivnih incidentov. V skladu s predhodnimi raziskavami rezultati podpirajo priporočila, da mora preprečevanje nasilja presegati uporabo ocenjevalnih instrumentov in usposabljanje zaposlenih ter vključevati kulturo varnosti in organizacijsko zavezanost preprečevanju ter obvladovanju agresivnega vedenja.

## 1 INTRODUCTION

Extant research shows that over 60% of healthcare workers experience violent behaviour over a one-year period, with verbal violence being the most common form; the highest rates are reported in North America and Asia, and the lowest in Europe (1, 2). Particularly in psychiatric settings, violence against healthcare workers is recognised as a major workplace and public health problem, causing stress, burnout, reduced productivity, and job dissatisfaction among employees (3, 4). Various types of violent behaviour occur, ranging from non-violent manifestations to severe physical violence, but most incidents are harmless, and serious injuries are rare (5,6,7,8). Verbal and psychological aggression by patients towards healthcare staff are the most common forms of aggression in psychiatric hospitals (6, 8,9,10). Aggression directed towards objects or the environment is also common and is often manifested as damage to furniture and equipment, but it can progress to physical aggression towards staff or patients (9, 11). Physical aggression most often manifests as pushing, grabbing, punching, and similar behaviours, often during medication administration or coercive measures (5, 7, 9, 12). Self-harm, such as hitting the head, cutting, strangling with ropes or straps, and hitting oneself, also frequently occurs in closed-door and intensive care units (8, 13). Verbal sexual harassment, such as unwanted sexual comments that are considered sexual violence, may be common against nurses, but physical sexual violence is rare (10). According to some studies, suicide occurs in 0.14–0.32% of all patients admitted to a psychiatric hospital, or 0.08–0.19% per admission (14).

The consequences of violent behaviour are multifaceted. For employees, they are manifested as increased stress, burnout, post-traumatic stress disorder, and a decision to leave the profession, while for patients, they are manifested as greater stigmatisation, a poorer therapeutic relationship, and lower quality of care received (15, 16). Due to the prevalence of violent behaviour and its associated consequences, its prevention should be a priority for health institutions. Effective prevention requires a multilevel approach that includes analyses of the existing situation, staff education, structured risk assessment, the use of de-escalation techniques, organisational support, and environmental and architectural adaptations (17,18,19). Key initial approaches include monitoring the situation and individual risk assessment for violent behaviour, especially when they allow for timely adaptation of preventive measures (20,21,22).

### 1.1 Purpose and objectives

The purpose of the study was to examine various forms of violent behaviour in patients with mental disorders at the University Psychiatric Clinic Ljubljana (UPC Ljubljana) during the period 2018–2021. The objective was to analyse the incidence and characteristics of violent behaviour in patients according to the gender of the perpetrator, the type of psychiatric ward, and the work shift. Aggressive incidents were documented using the OAS, while short-term violence risk was assessed using the BVC.

## 2 METHODS

A descriptive retrospective exploratory non-experimental empirical research design was employed.

### 2.1 Instrument description

The instrument consisted of three sections. In the first section, demographic data were recorded, hospital ward, date of assessment, time and duration of the event. For analytical purposes, the recorded time (23) of the event was categorised into morning (7 a.m.–2 p.m.), afternoon (2 p.m.–9 p.m.), and night (9 p.m.–7 a.m.) shifts, reflecting routine work shifts at the University Psychiatric Clinic Ljubljana. We divided the wards into three categories: acute psychiatric units, intensive care units, and other wards. The second section included the Overt Aggression Scale (OAS). The OAS was developed by Yudofsky et al. in the United States (23). It includes four categories of aggressive behaviour. We calculated the total aggression score (AS score) from the OAS following Silver and Yudofsky, with higher values indicating greater severity of expressed violence (24). The third section included the original six-item Brøset Violence Checklist (BVC). In addition, the local reporting form used at the University Psychiatric Clinic Ljubljana included one supplementary item (“aggressive behaviour towards people”), used solely for routine clinical documentation and not considered part of the original BVC risk assessment instrument. The BVC was developed by Lınaker & Busch-Iversen (25) and co-developed by Almvik in Norway (26). It includes six items: confusion, restlessness, tension, irritability, conflict, loudness, demandingness, verbal threats, physical threats, and violence towards objects. The answer options for each item are dichotomous (present: one point; not present: zero points). Although both instruments were incorporated into the same reporting form used in routine clinical practice, they served different clinical purposes. The BVC was used to assess short-term risk for violent behaviour and support preventive interventions, whereas the OAS was used to document and classify aggressive incidents that had already occurred. Therefore, the instruments were not treated as a single measurement tool but rather as complementary instruments assessing different aspects of violent behaviour.

For the BVC, we calculated a Cronbach's α coefficient (0.849) because the items measure a single concept predicting violent behaviour. For the OAS (both domains), we did not calculate a Cronbach's α because it does not represent a single scale of the same latent trait.

The research data supporting the findings of this study are openly available in the Zenodo repository (https://doi.org/10.5281/zenodo.20642670) (27).

### 2.2 Sample description

The sample included all duly completed OAS/BVC scales for the years 2018–2021. Each completed scale represented one unit of analysis. A total of 4,392 scales were completed over four years. The largest number of scales was completed for female subjects (n = 2372, 54.01%) in acute psychiatric units (n = 2572, 58.56%) and in the afternoon shift (n = 1927, 43.87%) (Table 1).

**Table 1:** Demographic data and other sample characteristics.

**Gender**	**n = 4392**	**%**
Male	2020	46.0
Female	2372	54.0
**Forms of aggressive behaviour**		
Verbal aggression	3695	84.1
Auto-aggressive behaviour	533	12.1
Aggressive behaviour towards objects	1449	33.0
Hetero-aggressive behaviour	2460	56.0
**Wards**		
Acute psychiatric unit	2572	58.6
Intensive care unit	973	22.2
Other wards	800	18.2
No data	47	1.1
**Shift**		
Morning (7 a.m. – 2 p.m.)	1611	36.7
Evening (2 p.m. – 9 p.m.)	1927	43.87
Night (9 p.m. – 7 a.m.)	758	17.3
No data	96	2.2

Legend: n – number; % – percentage

### 2.3 Description of the research process and data processing

Healthcare staff received regular training regarding the use of both instruments. Educational activities included face-to-face workshops, online educational sessions, annual feedback meetings, and case-based discussions focusing on the appropriate completion and interpretation of the OAS and BVC. The OAS/BVC scales have been routinely used at the University Psychiatric Clinic in Ljubljana since 2012. Data were entered into Microsoft Excel and analysed using IBM SPSS version 29.0 (IBM Corp., Armonk, NY, USA). Descriptive statistics and inferential analyses included Pearson's correlation coefficient, independent samples t-test, and one-way ANOVA with Bonferroni post-hoc tests. Effect sizes were measured using Cohen's d for comparisons between two groups and Eta-squared for comparisons involving three or more groups. P < 0.05 was considered statistically significant.

The data collection forms were generally completed in full. Missing data were limited to ward type (n = 47) and work shift (n = 96). Cases with missing information were excluded only from analyses requiring these variables, while all other available data were retained. No data imputation was performed because the proportion of missing values was small.

## 3 RESULTS

In terms of gender, auto-aggressive behaviour was significantly more frequent or more pronounced in women than in men (t = 4.319, p < 0.001). Men showed a higher level of hetero-aggressive behaviour compared to women (t = 13.409, p < 0.001). Verbal aggression was slightly, albeit significantly, more frequent in men (t = 3.293, p < 0.001). For hetero-aggressive behaviour, Cohen's d indicated an effect size of 0.41, which approached the threshold for a medium effect, whereas the effect sizes for the remaining outcomes were small or trivial (Table 2).

**Table 2:** Violent behaviour according to gender, type of ward, and work shift in the years 2018–2021.

**Gender-based violent behaviour**						

**Forms of aggressive behaviour**	**Gender**	**n**	**M**	**SD**	**t / p**	ES*
Auto-aggressive behaviour	male	192	0.36	1.13	4.319 / < 0.001	0.13
	female	341	0.52	1.30		
Aggression towards objects	male	689	0.89	1.31	1.759 / 0.079	0.05
	female	760	0.83	1.26		
Hetero-aggressive behaviour	male	1345	2.35	1.74	13.409 / < 0.001	0.41
	female	1115	1.63	1.78		
Verbal aggression	male	1675	2.13	1.40	3.293 / < 0.001	0.10
	female	2020	2.00	1.22		
AS score	male	2020	5.74	3.05	8.471 / < 0.001	0.26
	female	2372	4.98	2.83		

**Violent behaviour by type of ward**						

**Forms of aggressive behaviour**	**Ward**	**n**	**M**	**SD**	**F/p**	ES**
Auto-aggressive behaviour	acute psychiatric unit	270	0.38	1.13	74.827 / < 0.001	0.03
	intensive care unit	69	0.25	0.93		
	other wards	189	0.91	1.67		
	acute psychiatric unit	883	0.86	1.26		
Aggression towards objects	intensive care unit	346	0.94	1.32	5.627 / 0.004	<0.01
	other wards	207	0.74	1.30		
	acute psychiatric unit	1362	1.84	1.79		
Hetero-aggressive behaviour	intensive care unit	699	2.49	1.65	56.082 / < 0.001	0.03
	other wards	379	1.73	1.88		
	acute psychiatric unit	2219	2.06	1.28		
Verbal aggression	intensive care unit	851	2.23	1.22	23.287 / < 0.001	0.01
	other wards	582	1.81	1.43		
	acute psychiatric unit	2572	5.15	2.82		
AS score	intensive care unit	973	5.91	2.83	25.318 / < 0.001	0.01
	other wards	800	5.18	3.36		

**Violent behaviour by work shift**						

**Forms of aggressive behaviour**	**Work shift**	**n**	**M**	**SD**	**F/p**	ES**
Auto-aggressive behaviour	morning	1611	0.46	1.23	13.825 / < 0.001	0.01
	evening	1927	0.51	1.31		
	night	758	0.24	0.89		
Aggression towards objects	morning	1611	0.80	1.27	2.693 / 0.068	< 0.01
	evening	1927	0.90	1.31		
	night	758	0.87	1.24		
Hetero-aggressive behaviour	morning	1611	1.87	1.80	5.283 / < 0.001	< 0.01
	evening	1927	2.06	1.79		
	night	758	1.97	1.79		
Verbal aggression	morning	1611	2.03	1.31	2.356 / 0.095	< 0.01
	evening	1927	2.07	1.31		
	night	758	2.15	1.25		
AS score	morning	1611	5.16	2.90	8.322 / < 0.001	< 0.01
	evening	1927	5.55	3.03		
	night	758	5.23	2.77		

Legend: n – sample size; M – mean value; SD – standard deviation; t – t-test; F – ANOVA test; AS score – Total Aggression Score; p – statistical significance at p < 0.05; ES – effect size;

* Cohen's d;

** Eta-squared

The results for the forms of aggressive behaviour according to the type of ward and work shift are shown in Table 2. The Bonferroni post hoc test revealed significant differences among wards in the forms of aggressive behaviour. Auto-aggressive behaviour was significantly most common in other wards (open-door policy wards) compared to the acute psychiatric unit (p < 0.001) and the intensive care unit (p < 0.001). In aggression towards objects, the values for other wards were significantly lower than in the acute psychiatric unit (p = 0.040) and the intensive care unit (p = 0.003), between which no significant differences were observed. Hetero-aggressive behaviour was significantly most pronounced in the intensive care unit, where the values were higher compared to the acute psychiatric unit and other wards (p < 0.001 for both comparisons). The highest scores for verbal aggression were recorded in the intensive care unit (p = 0.002). AS score was also significantly higher in the intensive care unit than in the acute psychiatric unit and other wards (p < 0.001), but no significant differences were observed between the acute psychiatric unit and other wards. Eta-squared indicated small or trivial effect sizes for all outcomes.

Regarding work shifts, the Bonferroni post hoc test showed that auto-aggressive behaviour was significantly more frequent during the morning and evening shifts (p < 0.001). Hetero-aggressive behaviour was significantly more frequent in the evening shift (p = 0.003). The AS score was significantly higher in the evening shift than in the morning (p < 0.001) and in the night shift (p = 0.037). Eta-squared indicated small or trivial effect sizes for all outcomes.

The average values of BVC differed significantly across the period 2018—2021 (F = 10.287, p < 0.001). The lowest average value of BVC was recorded in 2020 (M = 4.61, SD = 1.58), and the highest in 2021 (M = 4.94, SD = 1.56). Across all years, the distribution of BVC scores was most frequent at 4, followed by 5 and 7 (Table 3). The Bonferroni post-hoc test showed that the mean BVC in 2021 was significantly higher than in other years (p < 0.001). Eta-squared resulted in a small effect size of 0.01.

**Table 3:** Comparison of BVC scores across years.

**BVC rating**		**0**	**1**	**2**	**3**	**4**	**5**	**6**	**7**	**Total**	**M**	**SD**	**F/p**
2018	n	0	11	26	68	91	41	10	112	359	4.68	1.83	10.287 / < 0.001
	%	0.00	3.06	7.24	18.94	25.35	11.42	2.79	31.20	100.00			
2019	n	0	10	53	230	317	252	60	295	1217	4.73	1.58	
	%	0.00	0.82	4.35	18.90	26.05	20.71	4.93	24.24	100.00			
2020	n	0	19	93	273	377	310	104	302	1478	4.61	1.58	
	%	0.00	1.29	6.29	18.47	25.51	20.97	7.04	20.43	100.00			
2021	n	0	8	45	202	348	252	125	358	1338	4.94*	1.56	
	%	0.00	0.60	3.36	15.10	26.01	18.83	9.34	26.76	100.00			
Total	n	0	48	217	773	1133	855	299	1067	4392	4.75	1.60	
	%	0.00	1.09	4.94	17.60	25.80	19.47	6.81	24.29	100.00			

Legend: n – number; % – percentage; M – mean; SD – standard deviation; F – ANOVA test; p – statistical significance at p < 0.05; Eta-squared = 0.01

A significant positive correlation was found between the AS and BVC scores (r = 0.672, p < 0.001).

**Figure 1:**
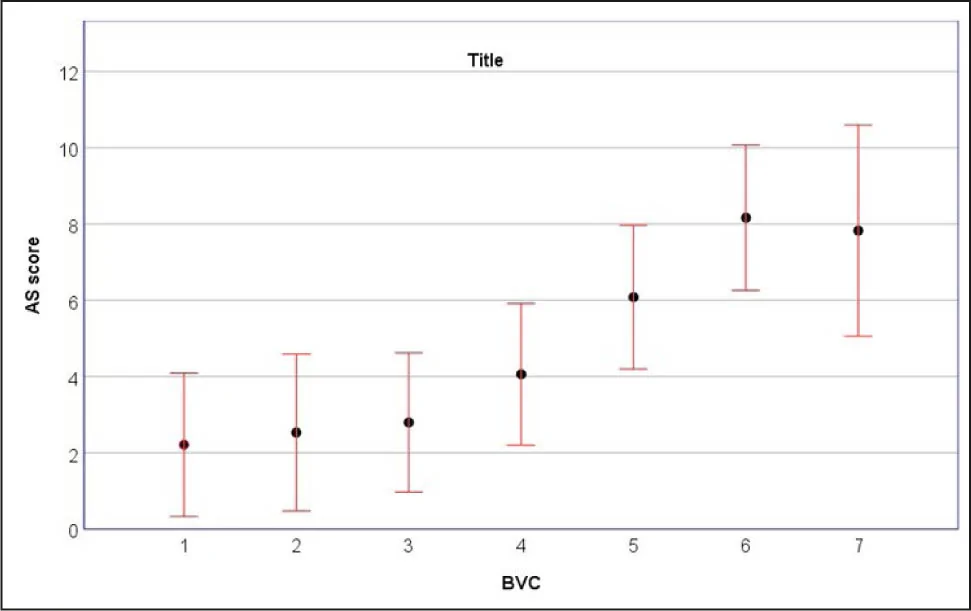
Mean values of the total aggression score (AS score) by BVC score.

Figure 1 shows the relationship between BVC score and AS score. The mean AS score increased gradually with increasing BVC score, with the lowest values recorded for low BVC scores and the highest for BVC scores of 6 and 7. The dispersion of AS score values increased with an increasing BVC score.

## 4 DISCUSSION

The most common form of violent behaviour in our study was found to be verbal violence (84.1%). Similar findings were reported by Čelofiga et al. (28) in a Slovenian national survey of acute psychiatric wards, where verbal aggression was likewise identified as the predominant form of aggressive behaviour. Physical violence was present in 56.0% of cases, which is consistent with foreign research findings (1, 2, 17, 29, 30). Violence against objects and auto-aggressive behaviour were present in our study at rates comparable to those reported in international studies (31,32,33).

The literature on gender differences in violent behaviour is inconsistent, with some studies reporting no significant differences (3, 28, 34) and others describing higher levels of violence among women in specific settings, particularly forensic psychiatry (35). In our study, women showed higher rates of auto-aggressive behaviour, consistent with previous findings (36), which may be related to the higher prevalence and severity of depression and anxiety among women (37,38,39). In contrast, men exhibited higher rates of verbal aggression, aggression towards objects, and hetero-aggressive behaviour, which may reflect differences in psychiatric diagnoses, substance use, personality traits, and responses to restrictive ward environments (6, 8, 40, 41).

The highest incidence of aggressive behaviour towards objects, other people, and verbal aggression was found in closed-door policy wards (acute psychiatric units and intensive care units). The risk of violent behaviour is more pronounced in patients treated in closed-door wards who are admitted involuntarily, which is also confirmed by foreign research findings (34, 42,43,44). Involuntary hospitalisation may be associated with feelings of coercion, loss of control, and restriction of personal freedom, which increases the likelihood of conflict and violent responses. An unexpected finding was the significantly higher level of auto-aggressive behaviour in open-door wards compared with acute psychiatric and intensive care units. Although self-harm has often been linked to psychological distress and restrictive psychiatric environments (45, 46), evidence comparing open and closed wards remains inconsistent (47). Therefore, the reasons for the observed pattern in our study require further investigation. One possible explanation is related to data collection procedures during the initial phase of monitoring, when a clear distinction between auto-aggressive behaviour and self-harming behaviour was not consistently applied.

Similarly to previous studies from other countries (35, 46), our study also showed that the evening shift had the highest incidence of violent behaviour, which may be related to staffing levels, less structured responsibilities, and other factors. Based on these findings, reducing aggressive incidents may require more structured afternoon activities, greater multidisciplinary team availability, and adequate staffing during high-risk periods (11). Differences between years should be interpreted cautiously, as the study period included the COVID-19 pandemic and the initial implementation phase of the violence monitoring system, both of which may have influenced reporting practices.

A strong correlation between BVC and AS scores was observed throughout the study period, reflecting the complementary roles of the BVC in assessing short-term violence risk (26) and the OAS in documenting aggressive incidents (23). The observed association supports the clinical usefulness of the BVC as an early warning tool for identifying patients who may require closer observation and timely preventive interventions. Because information on preventive interventions following elevated BVC scores was not available for analysis, the present study cannot determine whether aggressive incidents occurred despite appropriate preventive measures or because such measures were not implemented. However, risk identification alone does not guarantee prevention of aggressive incidents, as aggression is influenced by multiple patient, environmental, organisational, and clinical factors. The greater dispersion of AS scores at higher BVC values suggests that higher assessed risk does not always correspond to the same level of aggression, potentially reflecting the effects of de-escalation interventions and other preventive measures (48). Although correlation does not imply causation (49), the consistency of findings across several years supports the interpretation that the BVC identifies patients who require increased clinical attention and preventive interventions. Although several statistically significant differences were identified, effect size analysis indicated that most group differences were small in magnitude, suggesting limited practical significance. The largest effect was observed for hetero-aggressive behaviour between men and women (Cohen's d = 0.41). In contrast, the strong correlation between BVC and AS scores (r = 0.672) supports the clinical usefulness of the BVC instrument for assessing the risk and severity of aggressive behaviour, consistent with previous studies demonstrating its high predictive validity and utility in psychiatric inpatient settings (50, 51).

The findings highlight the continuing challenge of violence in psychiatric settings. Consistent with previous research, we support recommendations that violence prevention should be embedded within a broader culture of safety and organisational commitment. Interventions such as violence risk assessment, staff education, and de-escalation training are important but unlikely to achieve sustained effects when implemented in isolation (52,53,54).

Several limitations should be considered. Variability in the interpretation and completion of the OAS/BVC scales by healthcare professionals may have affected data consistency, and the number of completed assessments does not necessarily correspond to the number of patients or aggressive incidents. Due to the observational design, causal relationships cannot be inferred. In addition, part of the study period coincided with the COVID-19 pandemic, which may have influenced patient characteristics, admission patterns, staffing levels, and clinical practice. Furthermore, systematic violence monitoring began in 2018, and reporting compliance likely improved over time, potentially affecting the number of recorded incidents and completed assessments. Another limitation concerns the inclusion of a non-validated supplementary item in the local BVC reporting form, which requires cautious interpretation of findings associated with this adaptation. Furthermore, the OAS does not systematically capture contextual factors or intervention effectiveness, which were beyond the scope of this study.

## 5 CONCLUSION

Aggressive behaviour remains a significant challenge in psychiatric inpatient settings, with verbal aggression being the most common form. Differences were observed by gender, ward type, and work shift, and a strong association was found between BVC scores and the severity of aggressive incidents, as measured by the OAS. Together, the BVC and OAS provide complementary information on violence risk and aggressive behaviour. Despite its limitations, this study contributes to the limited evidence from Slovenian psychiatric settings and highlights the importance of systematic violence risk assessment. Consistent with previous research, sustainable violence prevention requires a culture of safety supported by risk assessment, staff education, and organisational commitment.
